# Associations between lifetime pregnancy and sexual risk behaviors among 15-24-year-old adolescent girls and young women in South Africa: Secondary analyses of the 2016 Demographic Health Survey

**DOI:** 10.1371/journal.pgph.0003317

**Published:** 2024-06-04

**Authors:** Ololade Julius Baruwa

**Affiliations:** Centre for Social Sciences Research (CSSR), University of Cape Town, Cape Town, South Africa; University of New South Wales - Kensington Campus: University of New South Wales, AUSTRALIA

## Abstract

Adolescent girls and young women (AGYW) in South Africa are highly vulnerable to HIV and poor sexual and reproductive health (SRH) outcomes. Interventions must respond to the unique needs of different AGYW groups, such as AGYW who have ever been pregnant. The objective of the study is to examine associations between pregnancy and sexual risk behaviors among AGYW in South Africa. This study used the 2016 nationally representative Demographic and Health Survey (DHS) of South Africa (n = 1935, 15–24 years old). Sexual risk behavior outcomes included: early sexual debut (defined as having sexual intercourse before the age of 15 years), age-disparate relationship (defined as having sexual partners who are five years and older in the past one month), multiple sexual partnerships, no condom use at last sex, and lastly, cumulative sexual risk (defined as reporting at least two of the outcomes: early sexual debut, age-disparate relationship, multiple sexual partners, and no condom use at last sex). Data analyses were conducted using logistic regression in STATA version 16. Statistical significance was determined at a P-value less than 0.05, with 95% confidence interval reported. AGYW who experienced lifetime pregnancy were more likely to report early sexual debut (OR = 1.71, 95%CI = 1.30–2.32), age-disparate relationships (OR = 1.58, 95%CI = 1.20–2.08), no condom use at last sex (OR = 2.77, 95%CI = 2.09–3.69), and cumulative sexual risk (OR = 1.82, 95%CI = 1.38–2.41). Multiple sexual partnerships showed no significant associations with lifetime pregnancy. Married or cohabiting AGYW were more likely to report cumulative sexual risk behaviors. (OR = 2.67, 95%CI = 1.91–3.71). Cumulative sexual risk behaviors were lower among AGYW with secondary education (OR = 0.57, 95%CI = 0.33–0.99) and those from rich households (OR = 0.62, 95%CI = 0.43–0.88). The findings underscore the need for interventions promoting safe sex and relationships, especially among AGYW who have experienced pregnancy. Programming should address the structural, socio-economic drivers of early pregnancy.

## Introduction

Adolescent girls and young women (AGYW) are confronted with unique sexual health challenges, including early pregnancy, unwanted pregnancy, and HIV, posing significant public health concerns [1]. In South Africa, where historical complexity and rich diversity shape the landscape, AGYW face health issues with substantial implications on their wellbeing. The prevalence of early pregnancies, affecting at least 40% of young girls [2], remains a major challenge exacerbated by socio-economic inequality [3], inadequate sexual education [4], and entrenched cultural norms [5].

Within the realm of early pregnancies lies the intricate issue of unwanted pregnancies, influenced by factors such as limited access to comprehensive family planning resources and sociocultural stigma surrounding contraception [6,7]. Additionally, AGYW in South Africa confront the persistent threat of HIV, with 38% of new infections occurring in those aged 15 to 24, particularly affecting females in this age group [8].

Considering the diverse and interconnected nature of the AGYW’s behaviors, culture, and socio-economic characteristics, it is crucial to examine the factors contributing to multiple sexual risks, which may lead to increased risks of unwanted pregnancy and HIV cases. This study aims to shed light on the diverse array of sexual risks that AGYW encounter, which include various behaviors that pose threats to the reproductive and general health of AGYW. Some of the sexual risk behaviors that AGYW engage in include age-disparate relationships, early sexual debut, multiple sexual partnerships, inconsistent condom use, and transactional sex, among others [4,9,10]. Designing focused interventions and public health strategies requires a thorough understanding of the prevalence of these risk factors. Studies have linked the experience of violence [11], lack of comprehensive sexual health education [12], and motherhood status [13] to multiple sexual risks among AGYW. However, there is a notable dearth of research concerning the relationship between lifetime pregnancy and multiple sexual risk behaviors among AGYW in South Africa. Additionally, there is insufficient exploration of how pregnancy status interacts with various sexual risk behaviors among AGYW. Moreover, there is a lack of comprehensive understanding regarding the factors contributing to the prevalence of pregnancy and sexual risk behaviors among AGYW, including the role of socio-economic factors.

To ensure overall health and provide a foundation for a healthier transition to adulthood, it is important to understand the risks AGYW face which are related to pregnancy and a variety of sexual risk behaviors. The aim of this study is therefore, to investigate the associations between pregnancy and sexual risk behaviors while controlling for other factors such as age, marital status, place of residence, education, wealth status, and employment status. This study endeavors to address the significant public health concerns related to pregnancy and various sexual risk behaviors among AGYW in South Africa. By investigating the associations between lifetime pregnancy and sexual risk behaviors, this study aims to provide valuable insights that can guide the development of evidence-based interventions and public health strategies tailored to the diverse challenges faced by AGYW. Further, this study seeks to fill the existing literature gaps by exploring the complex relationships between lifetime pregnancy and sexual risk behaviors among AGYW, contributing to a deeper understanding of the underlying factors that influence these behaviors. The findings from this study also aim to support AGYW in making informed decisions regarding their sexual health and overall well-being, thereby promoting their overall health and future opportunities.

## Methodology

This study utilizes data from the 2016 South Africa Demographic and Health Surveys (SADHS), focusing on women aged 15 to 49. From the initial pool of 9878 eligible female participants, 8514 were successfully interviewed, yielding an 86% response rate. Among these respondents, 1935 sexually active AGYW aged 15 to 24 were identified and included in the analysis.

### Ethics statement

This study utilized secondary data obtained from dhsprogram.org. Ethical considerations were observed during the original data collection, and written informed consent was obtained from the parents/guardians of the participants. Further details on the ethical aspects of the study can be accessed on the website at https://dhsprogram.com/data/dataset_admin/login_main.cfm.

### Variable measurements

#### Outcomes

This study investigated a series of sexual risk behaviors. The study explored sexual risk behaviors such as early sexual debut, age-disparate relationships, multiple sexual partners, no condom use at last sex, and cumulative sexual risk behaviors. Early sexual debut was defined as having sexual intercourse before the age of 15 years and measured as no and yes. The choice to establish the cut-off point for early sexual debut at 15 years is grounded in its alignment with international frameworks and guidelines, as outlined by esteemed sources such as the World Health Organization (WHO), along with corroborating findings from previous studies [14–16].

Age-disparate relationship refers to having sexual partners who are five years and older in the past 12 months. Multiple sexual partnerships were defined as sexual intercourse with more than one sexual partner in the past 12 months. No condom use at last sex was defined as a self-report of not using condoms with sexual partner(s) at last sex. Lastly, cumulative sexual risks were defined as having at least two of the outcomes: early sexual debut, age-disparate relationship, multiple sexual partners, and no condom use at last sex.

#### Independent variable

Lifetime pregnancy: Defined as currently pregnant, having had an abortion, or having given birth.

#### Socio-demographic variables

The selection of sociodemographic variables in this study was informed by previous research in the existing literature [9,17–19]. We measured six sociodemographic variables: age, education, married, or cohabiting, employment status, wealth index, and place of residence. Age was defined as the current age of the participant at the time of the study and was measured as 15–19 and 20–24 years. Education was defined as the highest level of education of AGYW at the time of the study and was measured as no education or primary, secondary, or higher education. Married or cohabiting as if married was defined as married or living with partners on or before the age of 24 and was measured as no or yes. Employment status was defined as the working status of AGYW at the time of the survey and was measured as not working or working. Place of residence was defined as the areas where AGYW lived at the time of the survey and was measured as urban or rural. Lastly, the wealth index was generated using the household items to classify AGYW into poor, middle, and rich categories.

### Data analysis

The analysis consisted of four steps. First, weighted frequencies of the analytic sample socio-demographic characteristics and outcomes were conducted. Second, a bar chart showing the prevalence of sexual risk behaviors by age of AGYW was plotted. Third, bivariate analyses of study outcomes and socio-demographic characteristics by lifetime pregnancy of AGYW using chi-square tests at p<0.05 level of significance were performed.

Fourth, the multivariable analysis used logistic regression models to investigate the associations between lifetime pregnancy and the outcomes of interest; early sexual debut, age-disparate relationship, multiple sexual partners, no condom use at last sex, and cumulative sexual risk behaviors.

Before the commencement of statistical analyses, data were weighted using the sampling weight to account for the differentials in the sample design and response rate. All data management and statistical analysis were performed using the Stata version 16. Results were interpreted using odds ratios. Statistical significance was declared if the P-value was less than 0.05 while the confidence interval was set at 95%.

## Results

### Characteristics of the study population

Table 1 presents the characteristics of the study population (n = 1935, 15–24 years). Findings revealed that 24.6% of AGYW started having sexual intercourse before the age of 15 years. Seven percent (6.9%) of AGYW have multiple sexual partners. Forty-five percent of AGYW did not use condoms at last sexual intercourse, and 39.3% were in age-disparate relationships.

**Table 1:** Study participants’ characteristics and sexual risk behavior outcomes of sexually active AGYW (15–24) years old AGYW in South Africa.

Variables	Sample size	Total n (%)	Never pregnant n (%)	Lifetime pregnancy n (%)	Chi-square, p-value
**Sexual risk behavior outcomes**					
**Early sexual debut** No Yes	1935	1467 (75.4) 468 (24.6)	625 (32.0) 169 (9.0)	842 (43.4) 299 (15.6)	0.0782
**Age-disparate relationship** No Yes	1754	1061 (60.7) 693 (39.3)	513 (29.1) 226 (12.7)	548 (31,7) 467 (26.6)	<0.001
**Multiple sexual partners (12 months)** No Yes	1935	1782 (93.1) 153 (6.9)	717 (37.8) 77 (3.2)	1065 (55.3) 76 (3.7)	0.2566
**No condom uses at last sexual intercourse.** No Yes	1754	957 (54.9) 797 (45.1)	512 (30.0) 227 (11.7)	445 (24.9) 570 (33.4)	<0.001
**Cumulative sexual risk behaviours** No Yes	1935	1337 (69.8) 598 (30.2)	609 (32.4) 185 (8.7)	728 (37.4) 413 (21.6)	<0.001
**Pregnancy status**					
Ever pregnancy No Yes	1935	794 (41.0) 1141 (59.0)	-	-	-
**Socio-demographic characteristics**					
Ever married or cohabiting No Yes	1935	1645 (82.5) 290 (17.5)	751 (38.8) 43 (2.2)	894 (43.7) 247 (15.3)	<0.001
Age 15–19 20–24 years	1935	652 (32.5) 1283 (67.5)	397 (20.3) 397 (20.8)	255 (12.2) 886 (46.8)	<0.001
Employment status Not working Working	1935	1692 (85.7) 243 (14.3)	706 (35.4) 88 (5.6)	986 (50.3) 155 (8.7)	0.6777
Place of residence Rural Urban	1935	904 (36.0) 1031 (64.0)	356 (13.6) 438 (27.5)	548 (22.4) 593 (36.5)	0.0566
**Education** No education/primary Secondary Higher	1935	121 (6.6) 1668 (84.5) 146 (8.9)	37 (2.0) 685 (34.9) 72 (4.2)	84 (4.6) 983 (49.7) 74 (4.7)	0.0783
**Wealth status** Poor Middle Rich	1935	943 (46.5) 458 (21.2) 534 (32.4)	340 (16.4) 191 (8.2) 263 (16.5)	603 (30.1) 267 (12.9) 271 (15.9)	<0.001

Weighted percentages

Fifty-nine percent of the study population had lifetime pregnancy. About two-thirds (67.5%) of the study population were between 20 and 24 years old, and 17.5% were married or cohabiting at the time of the survey. Sixty-four percent of the study population lived in urban areas, 85.7% were not working, 84.5% had secondary education, and 46.5% were from poor households.

### Prevalence of sexual risk behaviors by age group of AGYW

**Fig 1:**
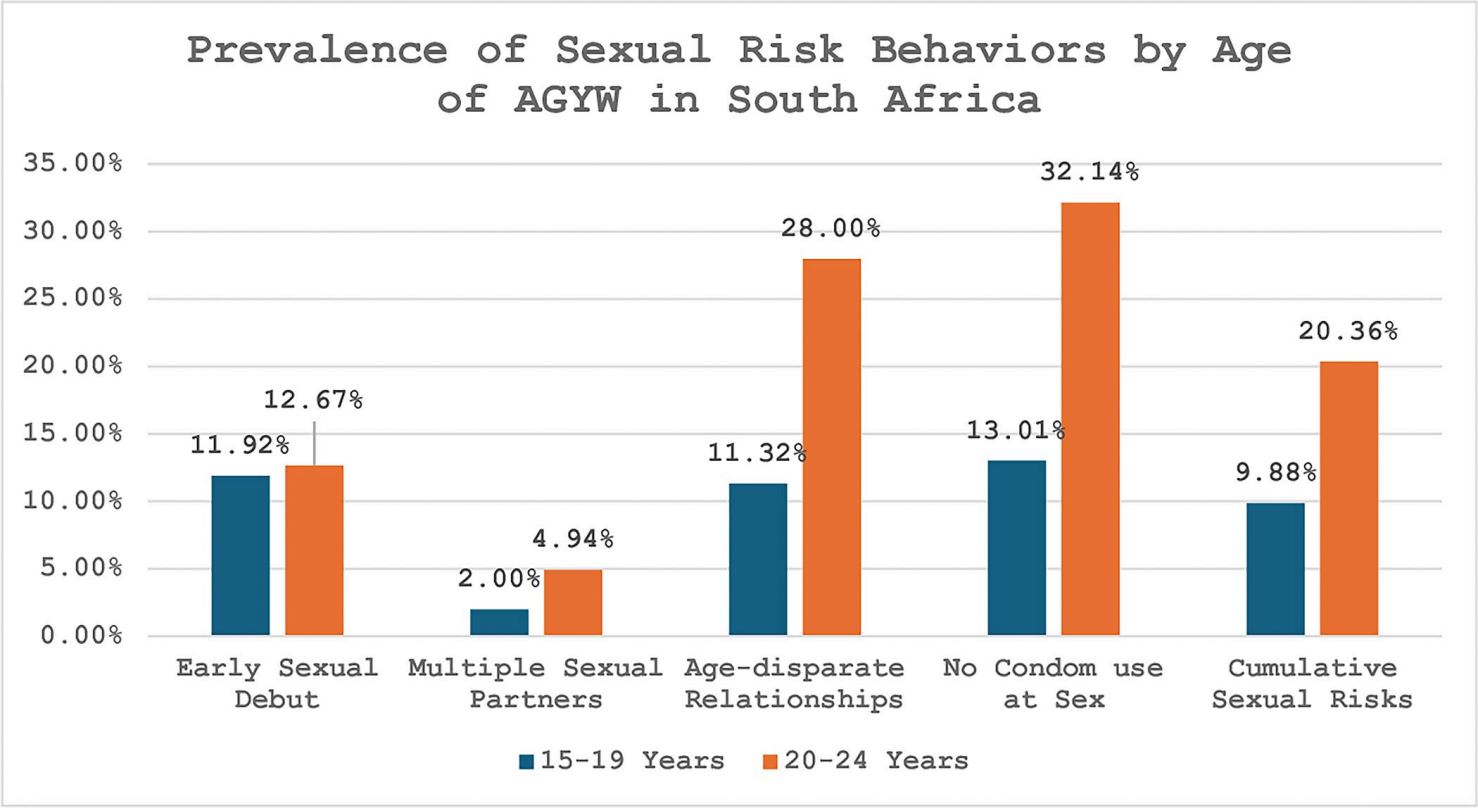
Prevalence of sexual risk behaviors by age of AGYW.

Fig 1 presents the prevalence of sexual risk behaviors among AGYW in South Africa by age group. The risk of early sexual debut was almost the same between AGYW who were between the ages of 20–24 years (12.67%) and those between the ages of 15–19 years (11.92%). Multiple sexual partnerships were more prevalent among 20–24 years AGYW (4.94%) compared to 15–19 years AGYW (2%). Concerning age-disparate relationships, the prevalence was 28% among 20–24 years AGYW compared to 11.32% among 15–19 years AGYW. Similarly, 20–24 years AGYW were more likely (32.14%) not to use condoms at last sex compared to 15–19 years AGYW (13.01%). Fig 1 also showed that there was a large disparity between 20–24 years AGYW (20.36%) and 15–19 years AGYW (9.88%) in terms of cumulative sexual risk behaviors.

### Bivariate associations between pregnancy status and HIV risk behaviors

Table 1 also presents the bivariate analysis. The bivariate analysis showed that age-disparate relationships, no condom use at last sexual intercourse, cumulated sexual risk behaviors, marriage or cohabitation, age, and wealth index had significant relationships with lifetime pregnancy.

The bivariate results showed that 31.6% of AGYW who experienced lifetime pregnancy started having sexual intercourse before the age of 15 years compared to 9% of those who were never pregnant. Approximately 26.6% of AGYW AGYW who experienced lifetime pregnancy engaged in age-disparate relationships, which was significantly lower among AGYW who were never pregnant (12.7%). In terms of multiple sexual partnerships, the prevalence of engaging in multiple sexual partnerships was 3.7% among AGYW who experienced lifetime pregnancy, and 3.2% among those who were never pregnant. Table 1 shows that among AGYW who experienced lifetime pregnancy, 45.1% did not use condoms at last sexual intercourse compared to 11.7% among AGYW who were never pregnant. Furthermore, 30.2% of AGYW engaged in cumulative sexual risk behaviors.

Regarding ever being married or cohabiting, 15.3% of AGYW who experienced lifetime pregnancy were married or cohabitating with their partners at the time of the survey. The prevalence of lifetime pregnancy was higher (46.8%) among 20–24 years compared to 15–19 years AGYW (12.2%). Fifty percent (50.3%) of AGYW who were not working had experienced lifetime pregnancy compared to 8.7% of AGYW who were working. Concerning place of residence, 36.5% of AGYW who experienced lifetime pregnancy lived in urban areas. For education, the prevalence of lifetime pregnancy was highest among respondents with secondary education (49.7%). Wealth status was significantly associated with lifetime pregnancy, with a higher prevalence among the poor (30.1%) compared to the middle (12.9%).

### Multivariable analysis of factors associated with sexual risk behaviors

Table 2 presents the odds ratios between lifetime pregnancy, and sociodemographic factors, and all the sets of outcomes (early sexual debut, multiple sexual partnerships, age-disparate relationships, and no condom use at last sexual intercourse).

**Table 2:** Logistic regression model showing the association between sexual risk behaviors and all the selected characteristics of AGYW in South Africa.

	Early sexual debut	Multiple sexual partners	Age-disparate relationships	No condom uses at last sexual intercourse	Cumulative sexual risk behaviors
	aOR (95% CI)	aOR (95% CI)	aOR (95% CI)	aOR (95% CI)	aOR (95% CI)
**Ever pregnancy** No Yes	1 1.71 (1.30–2.32)*	1 0.81 (0.53–1.25)	1 1.58 (1.20–2.08)*	1 2.77 (2.09–3.69)*	1 1.82 (1.38–2.41)*
**Ever married or cohabiting** No Yes	1 1.00 (0.69–1.47)	1 0.38 (0.18–0.80)*	1 2.01 (1.41–2.86)*	1 3.60 (2.47–5.24)*	1 2.67 (1.91–3.71)*
**Age** 15–19 20–24	1 0.34 (0.25–0.47)*	1 1.41 (0.95–2.08)	1 1.06 (0.81–1.38)	1 0.88 (0.69–1.14)	1 0.71 (0.55–0.90)
**Employment status** Not working Working	1 0.90 (0.57–1.40)	1 1.18 (0.64–2.20)	1 1.08 (0.75–1.55)	1 0.90 (0.62–1.30)	1 1.02 (0.70–1.50)
**Place of residence** Rural Urban	1 1.40 (1.02–1.91)*	1 0.76 (0.47–1.26)	1 0.90 (0.67–1.22)	1 0.69 (0.52–0.90)*	1 0.87 (0.66–1.15)
**Education** No education/primary Secondary Higher	1 0.55 (0.35–0.86)* 0.37 (0.16–0.83)*	1 0.96 (0.44–2.11) 1.09 (0.38–3.11)	1 0.80 (0.45–1.41) 0.79 (0.38–1.64)	1 0.40 (0.24–0.67) 0.50 (0.25–1.03)	1 0.57 (0.33–0.99)* 0.69 (0.32–1.47)
**Wealth status** Poor Middle Rich	1 0.87 (0.62–1.23) 0.69 (0.45–1.04)	1 1.23 (0.76–2.00) 1.00 (0.57–1.73)	1 0.89 (0.65–1.21) 0.81 (0.56–1.17)	1 0.90 (0.64–1.30) 0.82 (0.57–1.18)	1 0.85 (0.63–1.15) 0.62 (0.43–0.88)*

The multivariate analysis showed that AGYW who experienced lifetime pregnancy were more likely to have early sexual debut (OR = 1.71, 95%CI = 1.30–2.32). AGYW who were 20–24 years old were less likely to have early sexual debut (OR = 0.34; 95%CI = 0.25–0.47). AGYW who were living in urban residences were more likely to have had early sexual debuts (OR = 1.40; 95%CI = 1.02–1.91). AGYW with secondary and higher education were less likely to have engaged in early sexual debut (OR = 0.55; 95%CI = 0.35–0.86) and (OR = 0.37; 95%CI = 0.16–0.83), respectively.

Lifetime pregnancy showed no association with multiple sexual partners. However, ever being married or cohabiting and the age of AGYW were found to be associated with multiple sexual partners. The risk of multiple sexual partners was lower among ever-married or cohabiting AGYW (OR = 0.38; 95%CI = 0.18–0.80).

Regarding age-disparate relationships, the multivariate analysis showed that AGYW who experienced lifetime pregnancy were more likely to engage in age-disparate relationships (OR = 1.58; 95%CI = 1.20–2.08). Further, the analysis showed that the likelihood of engaging in age-disparate relationships was two times higher among ever-married or cohabiting AGYW compared to never-married AGYW (OR = 2.01; 95%CI = 1.41–2.82).

The risk of not using condoms at last sexual intercourse was higher among AGYW who experienced lifetime pregnancy (OR = 2.77; 95%CI = 2.09–3.69). The likelihood of not using condoms at last sexual intercourse was higher among AGYW who were married or cohabiting compared to never married AGYW (OR = 3.60; 95%CI = 2.68–4.82). The likelihood of not using condoms at last sexual intercourse was lower among AGYW living in urban residences (OR = 0.69, 95%CI = 0.52–0.90),

Concerning, cumulative sexual risk behaviors, the multivariate analysis showed that AGYW who experienced lifetime pregnancy were more likely to engage in cumulative sexual risk behaviors (OR = 1.82; 95%CI = 1.38–2.41). AGYW who were married or cohabiting were more likely to engage in cumulative sexual risk behaviors (OR = 2.67; 95%CI = 1.91–3.71). AGYW who had secondary education were less likely to engage in cumulative sexual risk behaviors (OR = 0.57; 95%CI = 0.33–0.99). Lastly, AGYW from rich households were less likely to engage in cumulative sexual risk behaviors (OR = 0.62; 95%CI = 0.43–0.88).

## Discussion

The aim of this research was to explore the correlation between lifetime pregnancy and sexual risk behaviors among adolescent girls and young women (AGYW) aged 15 to 24 in South Africa, utilizing data from the 2016 South African DHS. Specifically, the study investigated whether pregnancy status was linked to early sexual debut, multiple sexual partners, age-disparate relationships, condom non-use during last sexual intercourse, and cumulative sexual risk behaviors. Findings indicated that 59% of the study participants had experienced at least one pregnancy. Moreover, AGYW reporting lifetime pregnancy demonstrated higher likelihoods of early sexual initiation, lack of condom usage during last intercourse, engagement in multiple sexual partnerships, involvement in age-disparate relationships, and cumulative sexual risk behaviors.

### Factors associated with early sexual debut

The multivariate analysis in this study uncovered a noteworthy association, indicating that Adolescent Girls and Young Women (AGYW) with a history of pregnancy were more likely to experience early sexual debut. This is consistent with findings from prior studies [4,20], which established a link between early sexual debut and increased pregnancy rates among AGYW. This finding suggests that AGYW who experienced pregnancy at a young age may have initiated sexual activities earlier than their counterpart. Older AGYW aged 20–24 years exhibited a lower risk of early sexual debut, emphasizing the role of age in influencing sexual behaviors [9]. This association suggests that increased maturity, autonomy, and decision-making skills acquired over time may contribute to a more deliberate initiation of sexual activity. Furthermore, as AGYW enter their early twenties, a shift in priorities towards career and education might lead to delayed sexual activities, aligning with findings from McClinton Appollis et al., (2022) [9].

AGYW residing in urban areas demonstrated a heightened inclination toward early sexual debut, underscoring the influence of urbanization on sexual behaviors. While urban settings offer greater access to social and economic opportunities, including educational institutions, employment prospects, and exposure to media and technology, which can benefit AGYW, they also expose them to social pressures that encourage early sexual activity [21]. Consistent with previous studies [17,19], AGYW with secondary school and higher education demonstrated a lower tendency for early sexual debut. This association suggests that education plays a crucial role in increasing awareness and knowledge of sexual and reproductive health, empowering AGYW to make informed decisions. Additionally, educational attainment may influence socioeconomic factors, providing opportunities and supportive environments that contribute to a delay in sexual initiation [22].

### Factors associated with multiple sexual partners

Regarding multiple sexual partners, AGYW who were married or cohabiting exhibited a lower risk, reflecting the stability and dedication in such partnerships [23]. This finding aligns with cultural and societal norms that discourage extramarital sexual partnerships, emphasizing monogamy and commitment. However, AGYW aged 20–24 years were more likely to engage in multiple sexual partnerships compared to their younger counterparts, possibly due to the transitional phase into adulthood, increased independence, and expanded social networks. This aligns with previous studies indicating that this age group may be more inclined to explore relationships and sexuality, exposing them to a variety of potential partners [18,24].

### Factors associated with age-disparate relationships

AGYW who had ever been pregnant had a higher risk of involvement in age-disparate relationships. This association may be linked to factors such as a lack of comprehensive sexual education, limited reproductive health resources, or early sexual debut, increasing the likelihood of relationships with older partners. Additionally, socioeconomic factors like poverty or limited educational opportunities may contribute to pregnancies among AGYW, leading them to seek stability or financial support from older partners. AGYW who were married or cohabiting also showed a higher risk of age-disparate relationships, influenced by sociocultural norms and expectations. In some contexts, early marriages are acceptable, and there may be a relationship between marriage or cohabitation and economic factors, where AGYW seeking financial security are more likely to date older partners [18]. Power dynamics in such relationships may arise from difficulties in expressing themselves or negotiating safer sexual practices.

### Factors associated with no condom use at last sexual intercourse

Regarding no condom use at last sexual intercourse, AGYW who were ever pregnant exhibited higher risks, potentially due to the perception of trust in long-term or stable relationships like marriage or cohabitation [25]. Cultural or social norms regarding pregnancy and condom use may also play a role, with a belief that condom use is unnecessary or inappropriate in committed relationships, particularly during pregnancy. Similarly, married or cohabiting AGYW were more likely not to use condoms at last sexual intercourse, reflecting higher levels of trust and perceived intimacy in such relationships. The emphasis on stable relationships might be on family planning rather than disease prevention, contributing to reduced condom use.

This finding suggests that adolescent girls and young women (AGYW) residing in urban areas are less likely to engage in unprotected sexual intercourse compared to their counterparts in rural areas. Several factors may contribute to this trend. Urban environments often provide better access to sexual health resources such as clinics, education programs, and contraception services, which may increase awareness about the importance of condom use and safer sexual practices [26]. Additionally, urban areas typically have higher levels of education and socioeconomic status, which can correlate with increased knowledge about sexual health and greater access to condoms.

### Factors associated with cumulative sexual risks

The study revealed that AGYW who were ever pregnant had heightened risks of engaging in cumulative sexual risk behaviors, involving early sexual debut, multiple sexual partners, age-disparate relationships, or no condom use at last sexual intercourse. This points to complex interrelated factors making ever-pregnant AGYW more vulnerable, including socio-economic difficulties, limited access to necessary resources, unstable employment, and little opportunity for education. The experience of pregnancy may also influence relationships, self-perception, and mental health, impacting sexual behavior and decision-making. Furthermore, AGYW who had ever been married or cohabiting were more likely to engage in cumulative sexual risk behaviors. This association may be linked to socio-cultural and economic inequalities associated with marriage, limiting autonomy and decision-making power for AGYW. Marital or cohabiting status may contribute to a higher likelihood of engaging in cumulative sexual risk behaviors.

The multivariate analysis revealed that AGYW aged 20–24 years were less likely to engage in cumulative sexual risk behaviors compared to those aged 15–19 years. This association may be attributed to the greater developmental maturity of the older age group, as well as disparities in educational attainment. Older AGYW may have completed higher education, leading to improved health literacy and well-informed sexual behavior decisions. As AGYW enter their twenties, they might become more self-aware and base their decisions on personal values rather than peer pressure. The finding that AGYW with secondary education have a lower risk of engaging in cumulative sexual risk behaviors aligns with previous studies [27,28]. Secondary education is linked to increased knowledge, awareness, and decision-making abilities, making it a protective factor. AGYW with secondary education may have a better understanding of the risks associated with risky sexual behaviors, enabling them to make more informed decisions and potentially delaying the onset of sexual activity. Additionally, the study found that AGYW from wealthy households were less likely to engage in cumulative sexual risk behaviors, highlighting the role of higher socioeconomic status as a protective factor. Higher socio-economic status, often associated with wealthy households, can provide AGYW with greater access to resources and opportunities, discouraging engagement in risky behaviors. Financial independence may influence decision-making regarding sexual behaviors, with AGYW from rich households feeling more empowered to avoid cumulative sexual risk behaviors.

## Study limitations

While the present study sought to contribute to the existing body of knowledge, some limitations should be considered when interpreting the study’s results. First, the possibility of bias in reporting sexual behaviors due to social desirability. Second, recall bias regarding age at first sexual debut, especially among the older AGYW might have contributed to reporting bias. Third, due to the cross-sectional nature of the data, the findings of this study do not allow causal relationships. Lastly, some important sexual behavior variables, such as transactional sex and condom negotiation could not be included in the study due low sample size (response). These limitations notwithstanding, this study provides important insights into factors associated with sexual risk behaviors among AGYW in South Africa.

### The key implication of the findings

- Addressing the heightened risks of cumulative sexual behaviors among ever-pregnant AGYW calls for integrated interventions that consider the socio-economic challenges and limited access to resources faced by this group. Strategies should focus on empowering AGYW through educational opportunities, economic support, and mental health services to mitigate the complex interrelated factors contributing to sexual risk behaviors.
- Furthermore, the study suggests the significance of considering marital or cohabiting status in sexual health interventions for AGYW. Understanding the dynamics associated with age-disparate relationships and decreased condom use in stable relationships can inform targeted interventions promoting communication, negotiation skills, and safer sexual practices within partnerships.

## Conclusion

The findings of this study demonstrate concerning associations between pregnancy among AGYW and an increased likelihood of engaging in cumulative sexual risk behaviors. This study highlights the vulnerability of AGYW who have experienced pregnancy and suggests that this group may face additional challenges and situations that increase their propensity to engage in cumulative sexual risk behaviors. This highlights the importance of targeted interventions that address the specific needs and risks of ever-pregnant AGYW and ensure that comprehensive support mechanisms are in place to reduce the potential impact of risky sexual behaviors. Further, tailored HIV interventions that are specifically designed to address the risks associated with pregnancy and multiple sexual behaviors can effectively reach and support AGYW in lowering their risk of HIV infection as well as pregnancy. Interventions should also consider factors such as marital-related communication, decision-making authority, and access to reproductive health services to promote safer sexual practices and reduce the overall risk of adverse outcomes.

This study also demonstrates a significant relationship between cumulative sexual risk behaviors and socioeconomic factors such as education and wealth. The implication of these findings underscores the importance of socioeconomic factors in influencing the sexual behaviors of AGYW. These highlight equipping AGYW with knowledge and awareness, and provision of resources and economic empowerment may contribute to a reduced propensity of engaging in risky sexual behaviors.

## References

[1] Duby Z, McClinton Appollis T, Jonas K, Maruping K, Dietrich J, LoVette A, et al. As a young pregnant girl… the challenges you face”: exploring the intersection between mental health and sexual and reproductive health amongst adolescent girls and young women in South Africa. AIDS and Behavior. 2021 Feb;25:344–53. 32683636 10.1007/s10461-020-02974-3 PMC7368608

[2] Amoateng AY, Ewemooje OS, Biney E. Prevalence and determinants of adolescent pregnancy among women of reproductive age in South Africa. African Journal of Reproductive Health. 2022 Apr 24;26(1):82–91. doi: 10.29063/ajrh2022/v26i1.9 37585020

[3] Makiwane M, Gumede NA, Molobela L. Initiation of sexual behaviour and early childbearing: poverty and the gendered nature of responsibility amongst young people in South Africa. Journal of International Women’s Studies. 2018;19(5):209–26.

[4] Yakubu I, Salisu WJ. Determinants of adolescent pregnancy in sub-Saharan Africa: a systematic review. Reproductive health. 2018 Dec;15:1–1. 29374479 10.1186/s12978-018-0460-4 PMC5787272

[5] Mkwananzi S, Lebelo RS, Mashinini A, Ngake A, Paledi MS, Thwala LS. Socio-Cultural Predictors of Teenage Pregnancy in South Africa: A Cross-Sectional Study that Compares Rural and Urban Areas. Gender and Behaviour. 2022 Dec 1;20(4):20525–41.

[6] Coleman JN, Milford C, Mosery N, Choi KW, Greener LR, Matthews LT, et al. “I did not plan… that is what hurts”: Pregnancy intentions and contraceptive use among pregnant young women in KwaZulu-Natal, South Africa. African Journal of AIDS Research. 2021 Apr 3;20(2):149–57. 34003077 10.2989/16085906.2021.1914693 PMC9996636

[7] Haffejee F, Govender N, Reddy P, Sibiya MN, Ghuman S, Ngxongo T, et al. Factors associated with unintended pregnancy among women attending a public health facility in KwaZulu-Natal, South Africa. South African Family Practice. 2018 May 1;60(3):79–83.

[8] Zuma K, Simbayi L, Zungu N, Moyo S, Marinda E, Jooste S, et al. The HIV epidemic in South Africa: key findings from 2017 national population-based survey. International Journal of Environmental Research and Public Health. 2022 Jul 1;19(13):8125. doi: 10.3390/ijerph19138125 35805784 PMC9265818

[9] McClinton Appollis T, Jonas K, Beauclair R, Lombard C, Duby Z, Cheyip M, et al. Early sexual debut and the effects on well-being among South African adolescent girls and young women aged 15 to 24 years. International Journal of Sexual Health. 2022 Apr 3;34(2):242–53. 10.1080/19317611.2021.1979162 PMC9462400 36092761

[10] Toska E, Campeau L, Cluver L, Orkin FM, Berezin MN, Sherr L, et al. Consistent provisions mitigate exposure to sexual risk and HIV among young adolescents in South Africa. AIDS and Behavior. 2020 Mar;24:903–13. doi: 10.1007/s10461-019-02735-x 31748938 PMC7018679

[11] Orindi BO, Maina BW, Muuo SW, Birdthistle I, Carter DJ, Floyd S, et al. Experiences of violence among adolescent girls and young women in Nairobi’s informal settlements prior to scale-up of the DREAMS Partnership: Prevalence, severity and predictors. PloS one. 2020 Apr 22;15(4):e0231737. doi: 10.1371/journal.pone.0231737 32320405 PMC7176122

[12] Byansi W, Howell TH, Filiatreau LM, Nabunya P, Kaiser N, Kasson E, et al. Sexual health behaviors and knowledge among Ugandan adolescent girls: Implications for advancing comprehensive sexual health education technology. In child & youth care forum 2023 Dec (Vol. 52, No. 6, pp. 1227–1247). New York: Springer US. doi: 10.1007/s10566-023-09730-3 38031566 PMC10683936

[13] Cluver L, Rudgard WE, Toska E, Orkin M, Ibrahim M, Langwenya N, et al. Food security reduces multiple HIV infection risks for high‐vulnerability adolescent mothers and non‐mothers in South Africa: a cross‐sectional study. African Journal of Reproduction and Gynaecological Endoscopy. 2022 Aug 1;25(8):e25928. doi: 10.1002/jia2.25928 36008916 PMC9411725

[14] Mabaso M, Sokhela Z, Mohlabane N, Chibi B, Zuma K, Simbayi L. Determinants of HIV infection among adolescent girls and young women aged 15–24 years in South Africa: a 2012 population-based national household survey. BMC Public Health. 2018 Dec;18:1–7. 10.1186/s12889-018-5051-3 PMC5787232 29373958

[15] Lee RL, Yuen Loke A, Hung TT, Sobel H. A systematic review on identifying risk factors associated with early sexual debut and coerced sex among adolescents and young people in communities. Journal of clinical nursing. 2018 Feb;27(3–4):478–501. doi: 10.1111/jocn.13933 28639335

[16] World Health Organization. National AIDS programmes: a guide to indicators for monitoring and evaluating national HIV/AIDS prevention programmes for young people. 2004. Available: https://data.unaids.org/publications/irc-pub06/jc949-nap-youngpeople_en.pdf.

[17] Bengesai AV, Khan HT, Dube R. Effect of early sexual debut on high school completion in South Africa. Journal of biosocial science. 2018 Jan;50(1):124–43. doi: 10.1017/S0021932017000104 28416040

[18] George G, Beckett S, Reddy T, Govender K, Cawood C, Khanyile D, et al. Determining HIV risk for Adolescent Girls and Young Women (AGYW) in relationships with “Blessers” and age-disparate partners: a cross-sectional survey in four districts in South Africa. BMC Public Health. 2022 May 14;22(1):973. doi: 10.1186/s12889-022-13394-4 35568839 PMC9107706

[19] Sing’oei V, Owuoth JK, Otieno J, Yates A, Andagalu B, Smith HJ, et al. Early sexual debut is associated with drug use and decreased educational attainment among males and females in Kisumu County, Kenya. Reproductive health. 2023 Jul 27;20(1):111. doi: 10.1186/s12978-023-01639-3 37501066 PMC10375697

[20] Ahinkorah BO, Kang M, Perry L, Brooks F, Hayen A. Prevalence of first adolescent pregnancy and its associated factors in sub-Saharan Africa: A multi-country analysis. PloS one. 2021 Feb 4;16(2):e0246308. doi: 10.1371/journal.pone.0246308 33539394 PMC7861528

[21] Hailegebreal S, Gilano G, Seboka BT, Sidelil H, Awol SM, Haile Y, et al. Prevalence and associated factors of early sexual initiation among female youth in East Africa: further analysis of recent demographic and health survey. BMC women’s health. 2022 Jul 22;22(1):304. doi: 10.1186/s12905-022-01895-8 35869510 PMC9306043

[22] Amoateng AY, Baruwa O. Changes in the timing of sexual intercourse in Ghana: evidence from the demographic and health survey data, 1988–2014. African Population Studies. 2018;32(3).

[23] Baraka J, Lawson DW, Schaffnit SB, Wamoyi J, Urassa M. Why marry early? Parental influence, agency and gendered conflict in Tanzanian marriages. Evolutionary human sciences. 2022 Jan;4:e49. doi: 10.1017/ehs.2022.46 37588904 PMC10426069

[24] Makola L, Mlangeni L, Mabaso M, Chibi B, Sokhela Z, Silimfe Z, et al. Predictors of contraceptive use among adolescent girls and young women (AGYW) aged 15 to 24 years in South Africa: results from the 2012 national population-based household survey. BMC women’s health. 2019 Dec;19:1–7. 31830982 10.1186/s12905-019-0861-8 PMC6909538

[25] Matson PA, Fortenberry JD, Chung SE, Gaydos CA, Ellen JM. Weekly variations in feelings of trust predict incident STI within a prospective cohort of adolescent women from a US city. Sexually Transmitted Infections. 2018 Dec 1;94(8):594–7. doi: 10.1136/sextrans-2017-053431 29574464

[26] Hu L, Luo Y, Zhong X, Lu R, Wang Y, Sharma M, Ye M. Condom Use and Related Factors among Rural and Urban Men Who Have Sex With Men in Western China: Based on Information-Motivation-Behavioral Skills Model. Am J Mens Health. 2020 Jan-Feb;14(1):1557988319899799. doi: 10.1177/1557988319899799 ; PMCID: PMC7008563. 32028826 PMC7008563

[27] Gottfredson NC, Bhushan NL, Reyes HL, Pettifor AE, Kahn K. Effects of early social bonds on adolescent trajectories of sexual risk behaviors among south African girls. AIDS and Behavior. 2022 Apr;26(4):1173–82. doi: 10.1007/s10461-021-03472-w 34622349 PMC8940619

[28] Wambura M, Drake M, Kuringe E, Majani E, Nyato D, Casalini C, et al. Cash transfer to adolescent girls and young women to reduce sexual risk behavior (CARE): protocol for a cluster randomized controlled trial. JMIR research protocols. 2019 Dec 20;8(12):e14696. doi: 10.2196/14696 31859686 PMC6942193

